# Up-Regulation of Annexin-A1 and Lipoxin A_4_ in Individuals with Ulcerative Colitis May Promote Mucosal Homeostasis

**DOI:** 10.1371/journal.pone.0039244

**Published:** 2012-06-18

**Authors:** Linda Vong, Jose G. P. Ferraz, Neil Dufton, Remo Panaccione, Paul L. Beck, Philip M. Sherman, Mauro Perretti, John L. Wallace

**Affiliations:** 1 Hospital for Sick Children, Research Institute, Toronto, Ontario, Canada; 2 Farncombe Family Digestive Health Research Institute, McMaster University, Hamilton, Ontario, Canada; 3 Division of Gastroenterology, University of Calgary, Calgary, Alberta, Canada; 4 Centre for Biochemical Pharmacology, William Harvey Research Institute, London, United Kingdom; Duke University Medical Center, United States of America

## Abstract

**Background:**

One of the characteristics of an active episode of ulcerative colitis (UC) is the intense mucosal infiltration of leukocytes. The pro-resolution mediators Annexin-A1 (AnxA1) and lipoxin A_4_ (LXA_4_) exert counter-regulatory effects on leukocyte recruitment, however to date, the dual/cumulative effects of these formyl peptide receptor-2 (FPR2/ALX) agonists in the context of human intestinal diseases are unclear. To define the contribution of these mediators, we measured their expression in biopsies from individuals with UC.

**Methods:**

Colonic mucosal biopsies were collected from two broad patient groups: healthy volunteers without (‘Ctrl’ n = 20) or with a prior history of UC (‘hx of UC’ n = 5); individuals with UC experiencing active disease (‘active’ n = 8), or in medically-induced remission (‘remission’ n = 16). We assessed the mucosal expression of LXA_4_, AnxA1, and the FPR2/ALX receptor in each patient group using a combination of fluorescence microscopy, biochemical and molecular analyses.

**Results:**

Mucosal expression of LXA_4_ was elevated exclusively in biopsies from individuals in remission (3-fold, *P*<0.05 vs. Ctrl). Moreover, in this same group we observed an upregulation of AnxA1 protein expression (2.5-fold increase vs. Ctrl, *P*<.01), concurrent with an increased level of macrophage infiltration, and an elevation in FPR2/ALX mRNA (7-fold increase vs. Ctrl, *P*<.05). Importantly, AnxA1 expression was not limited to cells infiltrating the lamina propria but was also detected in epithelial cells lining the intestinal crypts.

**Conclusions:**

Our results demonstrate a specific up-regulation of this pro-resolution circuit in individuals in remission from UC, and suggest a significant role for LXA_4_ and AnxA1 in promoting mucosal homeostasis.

## Introduction

Ulcerative colitis (UC) is a relapsing disease characterized by periods of exacerbation and remission. One of the hallmarks of an active episode is the intense mucosal infiltration of lymphocytes, macrophages and polymorphonuclear leukocytes (PMN) [1]. These cells release an array of injurious mediators including proteases, cytokines and free radicals, resulting in edema, goblet cell depletion and extensive mucosal ulceration [2], [3]. Although recruited to extinguish a pro-inflammatory stimulus, there is emerging evidence that infiltrating leukocytes also release anti-inflammatory mediators to trigger resolution [4], [5]. Central to this paradigm is the existence of distinct pro-resolution circuits which modulate both the duration and intensity of the inflammatory response [6]. Their effectiveness is governed by the timely introduction and removal of various leukocyte subsets; for example, during an acute inflammatory response, resolution of inflammation is preceded by the replacement of PMNs with macrophages. At each step these cells act as a source, or contain the enzymatic machinery, for the synthesis of pro-resolution mediators. A deficiency of these ‘stop-signals’, exemplified in rodent models where such mediators are absent, results in a persistent and dysregulated inflammatory response [7], [8].

Annexin-A1 (AnxA1) is a calcium- and phospholipid-binding protein with potent anti-inflammatory activities. It is particularly abundant in cells of the host immune system, including monocytes, macrophages, and PMNs [9]. Functionally, AnxA1 attenuates leukocyte recruitment by inhibiting cell adhesion and transmigration [10], [11]. AnxA1-deficient mice display an increased susceptibility to dextran sodium-sulfate (DSS)-induced colitis, and an impaired recovery following withdrawal of DSS [12]. Important roles for AnxA1 in the regulation of mucosal regeneration and healing have also been reported [13].

Lipoxin A_4_ (LXA_4_) is a lipoxygenase (LO)-derived eicosanoid generated *in situ* by the sequential lipoxygenation of arachidonic acid during cell-cell interaction [14], [15]. LXA_4_ inhibits eosinophil and PMN trafficking, adhesion and transmigration [16], [17], and is strongly chemotactic for monocytes and macrophages [18], [19]; in the latter case the ability of LXA_4_ to stimulate the non-phlogistic phagocytosis of apoptotic PMNs [20], [21] defines its role as an innate modulator of resolution. Native LXA_4_, LXA_4_ analogues and aspirin-triggered lipoxins (ATL) inhibit PMN adhesion to intestinal epithelial cells [22], [23], as well as attenuate the secretion of chemokines from intestinal epithelia *in vitro* [24], [25].

Both AnxA1 and LXA_4_ activate and signal via a common receptor, the formyl peptide receptor 2/LXA_4_ receptor (FPR2/ALX) [26]. Individually, the counter-regulatory effects of AnxA1 and LXA_4_ on leukocyte recruitment are abolished following administration of FPR2/ALX antagonists [27]–[30], indicating the central role of this receptor in immune-regulatory responses. However, little is known regarding the dual or cumulative effects of these pro-resolution mediators in human pathological settings.

In this study, we examined the possibility that AnxA1 and LXA_4_ exert protective and/or reparative roles in human intestinal inflammation. Given that resolution is an active process, we hypothesized that a microenvironment which favors a resolution phenotype would drive an increase in levels of both these mediators. We demonstrate that LXA_4_ biosynthesis is elevated in mucosal biopsies from UC patients in medically-induced remission. Moreover, we observed in these same patients a concurrent up-regulation of AnxA1 in the mucosal lamina propria and intestinal epithelia, suggesting a reparative role for AnxA1 in healing of the intestinal mucosa. Together, these results are consistent with a significant role, and possibly concerted actions, for LXA_4_ and AnxA1 in promoting mucosal homeostasis.

## Results

### Proinflammatory cytokine and enzyme expression

The expression of mRNA for cytokines TNF-α, IFN-γ, and IL-1β, as well as COX-2 (Table 1), was significantly elevated in biopsies from individuals with active disease. These findings were consistent with the level of macroscopic inflammation described at the time of colonoscopy. In contrast, in biopsies from individuals in medically-induced remission the expression of all genes, but COX-2, was not significantly different from that in the healthy group.

**Table 1:** Pro-inflammatory cytokine expression.

	Healthy		UC	
mRNA expression (fold change)	Ctrl	Hx of UC	Active	Rem
TNF-α	1.00±0.46	1.09±0.26	4.77±1.67 *	1.19±0.21
IL-1β	1.00±0.47	3.57±0.66	7.87±1.41 *	1.36±1.36
IFN-γ	1.00±0.43	0.91±0.37	5.61±1.64 ^Ψ^*	1.19±0.28
COX-2	1.00±0.23	0.65±0.16	3.91±0.84 ^Ψ^	3.03±0.77 *

Quantitative RT-PCR analysis of pro-inflammatory cytokine and COX-2 expression in human colon biopsies. Assessment of TNF-α, IFN-γ, IL-1β and COX-2 mRNA levels in healthy subjects without (Ctrl) or with a prior history (Hx) of UC, UC patients with active disease (active), or UC patients in medically-induced remission. Data were normalized to β-actin gene expression (n = 5–20; *P<.05 vs. Ctrl, Ψ*P*<.05 vs. Hx of UC).

### Mucosal infiltration by granulocytes and macrophages

**Figure 1:**
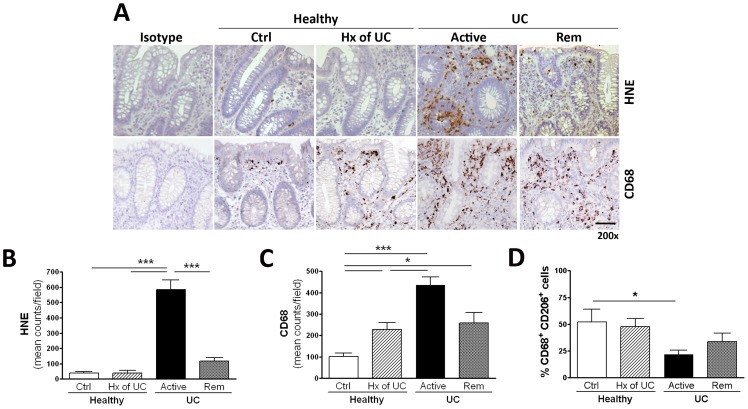
Expression of HNE and CD68 in colonic mucosal biopsies from healthy subjects without (Ctrl) or with a prior history (Hx) of UC, or UC patients with active disease (Active) or medically-induced remission (Rem) (**A**). HNE expression (Upper), used to detect PMNs was elevated in UC patients with active disease. CD68 expression (Lower), a pan macrophage marker, was elevated in active disease as well as in medically-induced remission. Cumulative analysis (mean counts/field) of HNE (**B**) and CD68 expression (**C**)**.** Dual-staining of macrophage populations with CD68 and CD206, markers for alternatively activated (M2) macrophages (**D**), Data are expressed as mean ± SEM (*P<.05, ***P<.001, n = 4). Magnification bar = 100μm.

Given the divergent roles of PMN and macrophages in the inflammatory response, we next assessed the infiltration of these cells into the colonic mucosa. PMN infiltration was significantly elevated in biopsies from patients with active disease, as assessed by HNE staining (Fig.1*A* *Upper* and Fig. 1*B*). The number of CD68^+^ cells, a pan macrophage marker, was increased in biopsies from patients with active disease, those in medically induced remission, as well as those with a prior history of colitis (Fig. 1*A* *Lower* and Fig. 1*C*). Dual-labeling of macrophages with CD68 and CD206 revealed a significant reduction in the percentage of alternatively activated macrophages in biopsies of those with active disease (Fig. 1D).

### Colonic LXA_4_ biosynthesis

**Figure 2:**
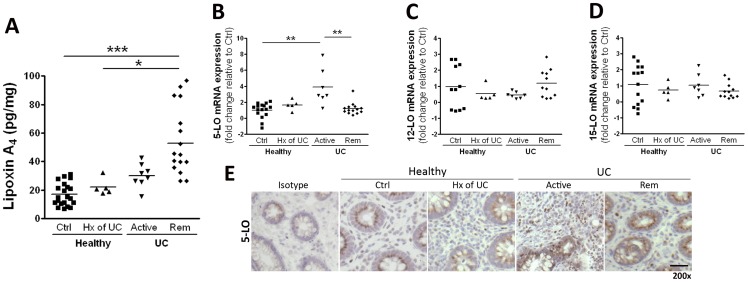
Colonic mucosal lipoxin A_4_ levels (**A**) in biopsies from healthy subjects without (Ctrl) or with a prior history (Hx) of UC, or UC patients with active disease (Active) or medically-induced remission (Rem). Lipoxin A_4_ levels were significantly elevated in the samples from patients who were in medically-induced remission compared with healthy subjects and those with a prior history of UC (>4 years disease-free). Quantitative RT-PCR analysis of 5-LO (**B**), 12-LO (**C**) and 15-LO (**D**) expression revealed an increase of 5-LO expression in patients with active disease. This correlated with an increase in the number of 5-LO-positive cells as assessed by immunohistochemistry (**E**). Data are expressed as fold change relative to Ctrl (*P<.05, **P<.01 ***P<.001, n = 5–20). Magnification bar = 100 μm.

LXA_4_ levels were significantly elevated (∼3-fold) in biopsies from patients in medically-induced remission (Fig. 2*A*) but not in any of the other groups. To assess for alterations in enzymes responsible for LXA_4_ biosynthesis, we first examined the mRNA expression of LO enzymes. 5-lipoxygenase (5-LO) mRNA expression was increased only in patients with active disease (Fig. 2*B*), with no significant changes in 12-lipoxygenase (12-LO) or 15-LO mRNA being identified (Fig. 2*C and 2D*, respectively). The increase in 5-LO mRNA expression was confirmed by immunohistochemistry (i.e., there was an increase in 5-LO^+^ cells in biopsies of patients with active disease) (Fig. 2*E*).

### AnxA1 expression in colonic mucosa

**Figure 3:**
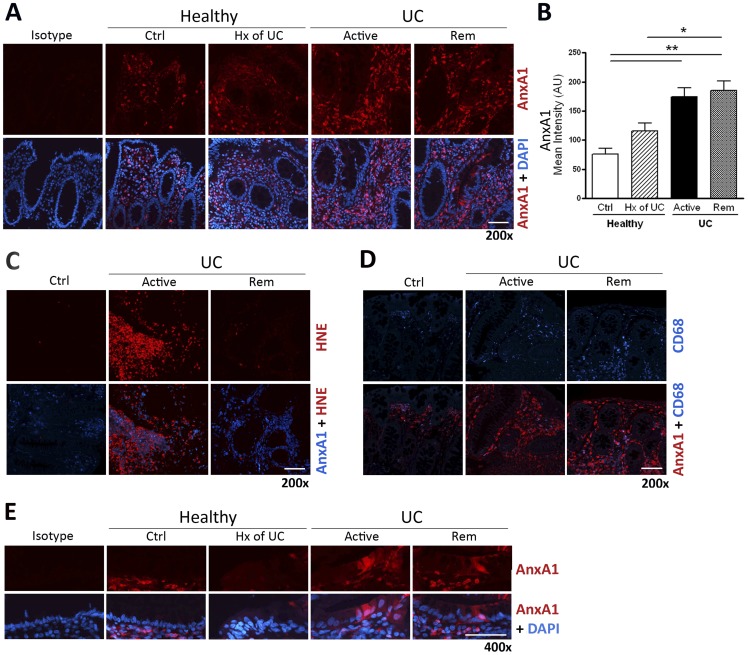
Expression of Annexin-A1 in colonic mucosal biopsies from healthy subjects without (Ctrl) or with a prior history (Hx) of UC, or UC patients with active disease (Active) or medically-induced remission (Rem) (**A**). Immunofluorescence detection of Annexin-A1 (red) demonstrates expression is increased in patients with UC, whether active or in medically-induced remission. Integrated pixel intensity revealed ∼3-fold increase in these groups, compared to healthy subjects (**B**). In biopsies from patients with active UC, Annexin-A1 (blue) staining could be localized to infiltrating PMNs (red; stained with anti-HNE) (**C**), however in biopsies from patients in medically-induced remission, Annexin-A1 staining (red) closely paralleled tissue infiltration by CD68^+^ macrophages (blue; stained with anti-CD68) (**D**). Annexin-A1 expression was also detected in crypt epithelial cells in subjects with UC, but not healthy subjects without/with a prior history of UC (**E**). Data are expressed as mean ± SEM (*P<.05, ***P<.001, n = 4). Magnification bar = 100 μm.

We used fluorescence microscopy to assess the expression and localization of AnxA1 in colonic mucosal biopsies. In healthy volunteers, a basal low level of AnxA1 expression was observed in cells of the intestinal lamina propria (Fig. 3*A, 3B*). In contrast, there was a significant increase in AnxA1 expression in biopsies from patients with active UC or those in medically-induced remission. Double-staining experiments revealed marked AnxA1 staining in neutrophils (co-localisation with HNE, a neutrophil granule protease) in biopsies of patients with active disease (Fig. 3*C*). In patients who were in medically-induced remission, AnxA1 expression was associated with macrophages, as evident from the paralleled expression with CD68 (Fig. 3*D*). Interestingly, AnxA1 immunostaining in biopsies from patients with UC (active or remission) was not limited to cells of the lamina propria, but appeared also in some of the epithelial cells lining the intestinal crypts (Fig. 3*E*). Such staining of epithelial cells was absent in biopsies from healthy volunteers.

**Figure 4:**
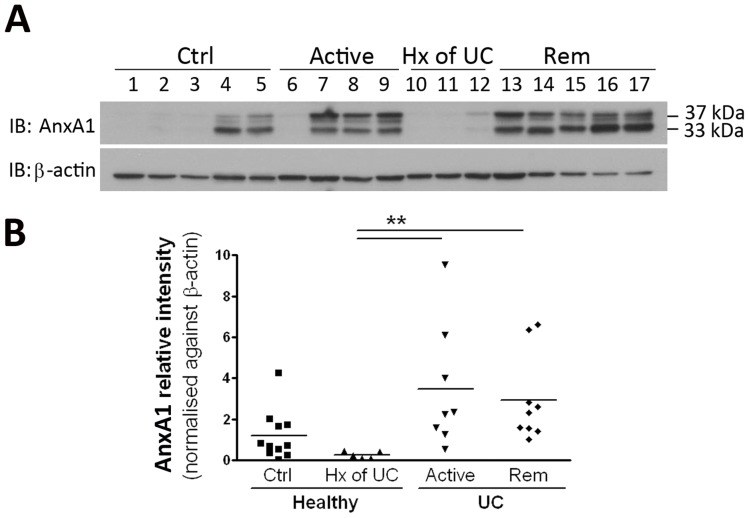
Detection of annexin-A1 NH_2_-terminal-intact and -cleaved fragments in biopsies from healthy subjects without(Ctrl) or with a prior history (Hx) of UC, or UC patients with active disease (Active) or medically-induced remission (Rem). Annexin-1 was overexpressed in colonic mucosal biopsies of patients with UC compared to healthy patients with a prior history of UC (**A**). Alongside the native 37 kDa fragment, a NH_2_-terminal cleaved isoform (33 kDa) could also be visualized, indicating specific degradation in response to externalization from activated cells. Analysis of relative intensity, where annexin-A1 immunoreactivity was normalized against β-actin (**B**). Data are expressed as mean ± SEM (**P<.01, n = 5–11).

Many of the biological effects of AnxA1 are mediated by the NH_2_-terminal domain [31], [32]. It is thought that proteolytic cleavage of the native 37 kDa protein results in termination of bioactivity, and cleavage-resistant AnxA1 mutants exhibit increased and prolonged anti-inflammatory activities [33]. Given the pro-inflammatory milieu in patients with active UC, we next assessed whether the expression of AnxA1 isoforms were altered among the patient groups. Biopsies from patients with active disease displayed a significant increase in AnxA1 protein expression, present as a characteristic NH_2_-terminal-cleaved 37/33kDa doublet (Fig. 4*A, 4B*). A similar increase was observed in patients in medically-induced remission, relative to healthy controls.

### Expression of FPR2/ALX receptor

**Figure 5:**
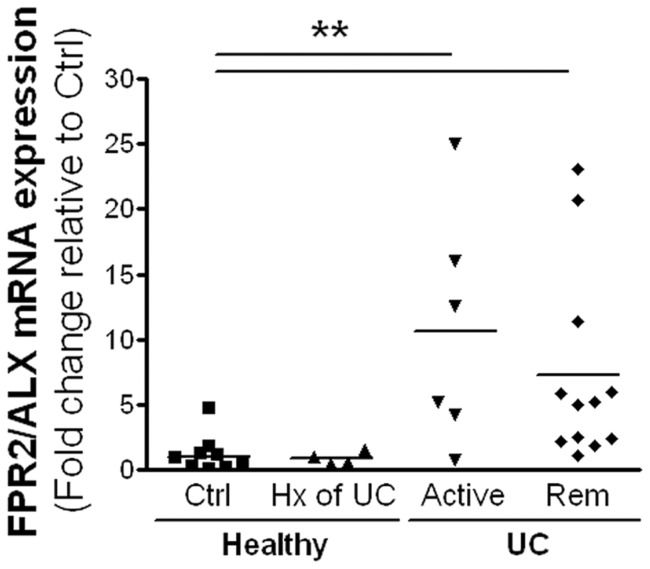
mRNA expression of FPR2/ALX in colonic mucosal biopsies from healthy subjects without(Ctrl) or with a prior history (Hx) of UC, or UC patients with active disease (Active) or medically-induced remission (Rem). Increased expression of FPR2/ALX was detected in biopsies from individuals with UC. Data are expressed as fold change relative to Ctrl (**P<.01, n = 5–12).

In line with colonic mucosal levels of AnxA1, analysis of FPR2/ALX mRNA expression revealed an increase in expression in biopsies from patients with active disease (10.7±3.6-fold, *P*<.05) as well as in those in medically-induced remission (7.3±2.9-fold, *P*<.05), relative to healthy controls (Fig. 5).

## Discussion

Much evidence supports the capacity of infiltrating leukocytes to synthesize, either individually or cooperatively, a number of anti-inflammatory and pro-resolving factors [34]. In the present study, we set out to document the contribution of a distinct pro-resolution circuit, namely the liberation and actions of AnxA1 and LXA_4_, in individuals with active or remittent UC.

Transepithelial migration of PMN from the microcirculation to the mucosa results in impaired barrier function and destruction of tissues [35], [36]. Consistent with the literature, we observed PMN infiltration in colonic biopsies taken from individuals with active UC, but not in the other patient groups. Alongside the elevation in pro-inflammatory cytokine transcript levels (TNF-α, IFN-γ and IL-1β) and COX-2 mRNA expression, the local infiltration by cell types such as monocytes and macrophages likely contributes to this inflammatory milieu [37]. Intestinal macrophages play a fundamental role in host defense, including the phagocytosis and killing of microorganisms. Blood monocytes recruited to the inflamed mucosa do not display the same level of tolerogenicity as their resident counterparts; instead, these cells retain or have increased inflammatory capabilities [38]. Consistent with the high numbers of PMN in biopsies from individuals with active UC, we also observed an increase in infiltration of CD68^+^ macrophages. Only a small proportion of these cells were of the M2 phenotype, as demonstrated by dual-labeling of macrophages with CD68 and CD206. However, the abundance of macrophages in biopsies from individuals in medically-induced remission, where there was an absence of PMN infiltration and an increase in COX-2 transcript levels, likely denoting an environment that is more conducive to the resolution of mucosal inflammation.

In a rodent model of self-resolving peritonitis, analysis of macrophage phenotypes, specifically during the resolution phase, revealed a new class of resolution-phase macrophages that share markers of both classically activated and alternatively activated cells [39]. These resolution-phase macrophages synthesize high levels of COX-2 as well as the anti-inflammatory mediator PGD_2_. We noted an increase in the number of infiltrating macrophages in biopsies from individuals in long-term remission, previously reported to be high producers of PGD_2_ [40].

Given their roles in host defense, invading leukocytes are often viewed as pro-inflammatory. However, there is accumulating evidence that such cells synthesize distinct counter-regulatory mediators that promote the resolution of mucosal inflammation. Previous studies have documented the inhibitory effects of LXA_4_ on leukocyte adhesion and transmigration [41], [42], and LXA_4_ analogues have also been shown to accelerate resolution in rodent models of colitis [43], [44].

The results from our study identify a significant increase in LXA_4_ synthesis exclusively in biopsies from patients in medically-induced remission. This further supports the hypothesis that there exists a pro-resolution microenvironment in the intestinal mucosa of individuals in remission from UC, and may be a means through which recruited macrophages coordinate the clearance of PMN. LXA_4_ synthesis has been reported in pulmonary disease [45], periodontitis [46] and nasal polyps [47]. We observed an increase in 5-LO expression, at the transcript and protein levels, in biopsies taken from individuals with active UC. This is consistent with known increases of leukotriene-dependent enzymes in active IBD [48]. However, we found no changes in LO expression in biopsies obtained from individuals in medically-induced remission. Thus, the observed increase in mucosal LXA_4_ synthesis likely stems from an augmentation of LO enzyme activity, rather than enzyme expression.

A new view regarding mechanisms of inflammatory resolution emerged when the short-lived lipid LXA_4_ and the glucocorticoid-regulated protein AnxA1 were shown to share the same receptor target (FPR2/ALX) [49]. This indicates the existence of a convergence between specific effectors for theresolution of inflammation. Of interest, both LXA_4_ and AnxA1 mediate IL-10-dependent inflammatory hyporesponsiveness in a model of intestinal ischemia/reperfusion injury [50]. Blockade of FPR2/ALX, LXA_4_ production, and the use of neutralizing AnxA1 antibodies result in an increase in tissue injury, TNF-α production and lethality. A direct functional association between LXA_4_ and endogenous AnxA1 has recently been described in human resting PMN *in vitro* [51]. However, the association between LXA_4_ and AnxA1 has not been studied in man and, more importantly, in human pathological settings. Thus, alongside LXA_4_ synthesis, we determined the expression and proteolysis of the AnxA1 in intestinal human samples.

Examination of colonic perfusates from UC patients indicates that AnxA1 secretion may be dependent on the severity of inflammation [52]. In the present study, we observed AnxA1-expression in the colonic mucosa of healthy individuals and those with UC, although expression was significantly elevated in the latter group. Whereas AnxA1 was localized predominately to PMN in biopsies of individuals with active disease, during disease remission AnxA1 expression switched to CD68+ macrophages. We speculate that high AnxA1 levels may be a characteristic of the recently described pro-resolving macrophages, at least in pre-clinical and clinical models of IBD, though future studies will be needed to specifically address this hypothesis. It is of interest to note that an increased susceptibility, mucosal injury, and clinical morbidity is observed in AnxA1-deficient mice administered dextran sodium sulfate (DSS) [53]. This dysregulated inflammatory response is compounded by an abated recovery following withdrawal of DSS administration, thereby providing strong proof-of-concept to the pro-resolving nature of AnxA1 in gut inflammation.

There is accumulating evidence that AnxA1 may also enhance mucosal healing. Peptides based on the NH_2_-terminal region of AnxA1 stimulate epithelial cell migration [54], which is an important step in the restitution and wound healing process. Gastric mucosal repair of acetic acid-induced ulcers is significantly impaired in AnxA1-deficient mice, whereas in wild-type littermates healing of the ulcer region is accompanied by an increase of AnxA1 at the ulcer margin [55]. Rodents administered DSS also display an increase of AnxA1 in both surface and crypt epithelial cells [56]. When assessing colonic biopsies for the formation of cleaved AnxA1 products by western blotting, we observed a similar elevation of AnxA1 expression in biopsies from individuals with UC, relative to those in the healthy group. In each case, AnxA1 was present as a 37/33kDa doublet, corresponding to the NH_2_-terminal-intact and -cleaved species, respectively [57].

Another interesting observation was the up-regulation of AnxA1 in intestinal epithelial cells of biopsies from individuals with active UC or in medically-induced remission. The increase of AnxA1 was diffuse, and not present in all crypt epithelial cells, which we speculate is likely reflective of the degree of mucosal inflammation and rate of cell turnover at any one point in time. This finding may be further evidence of the reparative roles of AnxA1 in the context of intestinal mucosa, independent of infiltrating leukocytes. It is noteworthy though that stimulation of epithelial cell motility, while important for mucosal healing, may also have pathophysiological consequences if dysregulated. In line with this view, an increase of AnxA1 expression has been associated with the development of tumor metastasis and colonic adenocarcinoma [58]–[60].

As mentioned above, the effects of AnxA1 and LXA_4_ are mediated via a common G_i_-protein-coupled receptor, namely FPR2/ALX [61]. FPR2/ALX is one of a family of pertussis toxin-sensitive FPR receptors [62] that interact with structurally diverse pro-and anti-inflammatory ligands [63]. The actions of AnxA1 and LXA_4_ are abolished with the use of FPR2/ALX antagonists [64]–[70] as well as in FPR2/ALX-deficient mice [71], highlighting the fundamental role of this receptor in transducing non-redundant anti-inflammatory signals. We demonstrate herein the up-regulation of FPR2/ALX mRNA in biopsies of UC patients in medically-induced remission. The concurrent up-regulation of this receptor, alongside the observed increase in synthesis of the pro-resolution mediators AnxA1 and LXA_4_ is consistent with a microenvironment that favors mucosal homeostasis. LXA_4_-dependent ligation of FPR2/ALX down-regulates epithelial secretion of the chemokine CXCL-8 [72], which likely contributes to the reduced inflammation observed in biopsies obtained from patients in medically-induced remission, compared to those with active disease. No changes in FPR2/ALX expression were observed in biopsies taken from patients with a prior history of UC (long-term, medication-free remission for >4 years), perhaps indicating other pro-homeostatic mechanisms are in effect [73].

In summary, this study has documented an increase in mucosal synthesis of two pro-resolution mediators, AnxA1 and LXA_4_, in individuals in medically-induced remission from UC. The concerted up-regulation of both ligand and receptor, in a previously pro-inflammatory setting, indicates a switch to a microenvironment that is conducive to the resolution of inflammation. Moreover, in the same individuals there was an up-regulation of AnxA1 synthesis by intestinal epithelial cells, consistent with its role in mucosal repair. We propose that this short-lived lipid and protein/peptide pair act in concert to safeguard effective inflammatory resolution. It is likely that parallel or converging pro-resolution networks, exemplified by the LXA_4_/AnxA1 pair, are activated in multiple inflammatory settings, and is a scenario that could become paradigmatic for other mediators of inflammation.

## Materials and Methods

### Ethics Statement

This study was approved by the Ethics Committee at the University of Calgary. Each patient gave their written consent prior to participation in this study and all experiments were conducted according to the principles expressed in the Declaration of Helsinki.

### Patients and tissue samples

Colonic mucosal biopsies were obtained from two broad patient groups: healthy individuals undergoing colonoscopy for routine colon cancer screening, and individuals with UC. These groups were subdivided; for healthy individuals, samples were obtained from those with no history of UC (‘control’ group; n = 8 male and 12 female; mean age 51±9 years), or those diagnosed previously with UC but had not experienced any bout of disease nor required any medication for UC for at least 4 years (‘prior history of UC’ group; n = 5 female; mean age 47±11 years). Patients with UC were divided into two groups: those with active disease (‘active’ group; n = 5 male and 3 female; mean age 43±16 years), and those in clinical and endoscopic remission while on maintenance therapy with either oral/topical 5-aminosalicylic acid or immunosuppressive/ biological therapy (‘remission’ group; n = 9 male and 7 female; mean age 44±13 years). Details regarding patient characteristics, such as gender, age, and clinical activity were obtained from medical records. Mucosal biopsies were taken from the left colon in close proximity to biopsies used for histological assessment. Samples intended for quantitative PCR, LXA_4_ measurement, or western blotting were stored at −80°C until ready for processing. Samples for immunohistochemistry were fixed in 10% neutral-buffered formalin.

### Quantitative PCR

Total RNA from colonic biopsies was extracted using the RNeasy Mini Kit (Qiagen, Valencia, CA) and two-step quantitative PCR was performed, as described [74]. Bioinformatic-validated high efficiency primer assays for human TNF-α (NM_000594), IL-1β (NM_000576), IFN-γ (NM_000619), COX-2 (NM_000963), 5-LO (NM_000698), 12-LO (NM_000697), 15-LO (NM_001140), FPR2/ALX (NM_001462) and β-actin (NM_001101) were obtained from Qiagen (Valencia, CA). All data were analyzed using Ct values obtained from Realplex software (Eppendorf, Ontario, CA), and amplification and relative quantification of gene products determined using the ΔΔC_t_ method, where target genes were normalized against the housekeeping gene β-actin.

### Western blotting

Tissue samples were processed and proteins separated by 12% SDS-PAGE, as described previously [75]. Primary polyclonal rabbit anti-AnxA1 (Invitrogen) and monoclonal mouse anti-β-actin (Sigma Aldrich) antibodies were used. Secondary anti-rabbit and anti-mouse IgG antibodies were conjugated to horseradish peroxidase (GE Healthcare), and visualized using enhanced chemiluminescence (ECL) detection kit (GE Healthcare) on a Chemi-doc gel imaging system (Bio-Rad). Densitometric analysis was performed using ImageLab 2.0 software (Bio-Rad, Ontario, CA), with AnxA1 protein normalized against β-actin expression.

### Histology and immunohistochemical analysis

Colonic biopsies from four patients per group were used for immunohistochemistry. For analysis of HNE and CD68 (markers of PMNs and macrophages, respectively), or 5-LO expression, sections were incubated in 3% H_2_O_2_ for 15min and then steamed for 30min in 10mM citrate buffer (pH 6.0)/0.05% Triton X-100. Sections were blocked in 10% normal serum, and incubated with mouse anti-human HNE (clone NP57, DAKO Cytomation), mouse anti-human CD68 (clone PG-M1, DAKO), mouse anti-human CD206 (ABCAM) or rabbit anti-5-LO (Cayman Chemical) overnight at 4°C. Sections were washed in PBS, incubated with anti-mouse or anti-rabbit biotinylated secondary antibodies (Vector Laboratories), and visualized by avidin-biotin-peroxidase detection using the Vectastain Elite ABC kit (Vector Laboratories). 3–3′ diaminobenzidine (DAB; Vector Laboratories) was used as the chromagen, and sections counterstained with Mayer's hematoxylin (Sigma Aldrich). For immunofluorescence detection of AnxA1, sections were blocked with 3% BSA and incubated with a monoclonal antibody raised against full-length human AnxA1 (mAb1B [76]) overnight, 4°C. To examine co-localization of AnxA1 with HNE or CD68, rabbit anti-AnxA1 antibody (Invitrogen) was incubated alongside anti-HNE or anti-CD68 antibodies. Sections were washed and incubated with AlexaFluor®568 goat anti-mouse IgG, AlexaFluor®350 goat anti-mouse IgG, AlexaFluor®568 goat anti-rabbit IgG, or AlexaFluor®350 goat anti-rabbit IgG (Invitrogen). To examine co-localization of CD68 with CD206, sections were incubated with AlexaFlour®568 goat anti-mouse and AlexaFluor®488 goat anti-mouse, respectively. Sections were then mounted with ProLong Gold containing DAPI (Invitrogen) or Vectashield fluorescence mounting medium (Vector Laboratories). Fluorescence was visualized on a Nikon Eclipse 80i microscope (Nikon) equipped with a DS-QiMc monochromatic camera (Nikon) and X-Cite® Series 120Q Xenon lamp. NIS-Elements BR3.1 software (Nikon) was used for all analyses. Images were recorded at identical gain settings, and mean intensity calculated per image field. Four image fields were taken of each section.

### Lipoxin A_4_ measurement

Measurement of LXA_4_ was performed, as previously described [77], using a commercially available LXA_4_ ELISA kit (Oxford Biomedical Research, Canada). Briefly, colonic biopsies were homogenized in extraction buffer (isopropranol/ethanol/0.1N HCl; 3∶3∶1), and diluted 1∶1 with deionized water. The sample was centrifuged at 1,500*g* for 10min at 4°C, the organic phase transferred to a new tube, acidified to pH 3.5 and purified on pre-conditioned C18 Sep-Pak light columns (Millipore). The eluate was evaporated to dryness under a gentle stream of nitrogen gas and levels of LXA_4_ determined using a commercially available LXA_4_ ELISA kit (Oxford Biomedical Research, Canada).

### Statistical analysis

Data are presented as mean±SEM. Comparisons among groups of data were made using a one-way ANOVA followed by the Kruskal-Wallis test. An associated probability (*P*<.05) was considered significant.

## References

[1] Xavier RJ, Podolsky DK (2007). Unravelling the pathogenesis of inflammatory bowel disease.. Nature.

[2] Grisham MB (1994). Oxidants and free radicals in inflammatory bowel disease.. Lancet.

[3] Nusrat A, Parkos CA, Liang TW, Carnes DK, Madara JL (1997). Neutrophil migration across model intestinal epithelia: monolayer disruption and subsequent events in epithelial repair.. Gastroenterology.

[4] Levy BD, Clish CB, Schmidt B, Gronert K, Serhan CN (2001). Lipid mediator class switching during acute inflammation: signals in resolution.. Nat Immunol.

[5] Spite M, Norling LV, Summers L, Yang R, Cooper D (2009). Resolvin D2 is a potent regulator of leukocytes and controls microbial sepsis.. Nature.

[6] Serhan CN, Savill J (2005). Resolution of inflammation: the beginning programs the end.. Nat Immunol.

[7] Babbin BA, Laukoetter MG, Nava P, Koch S, Lee WY (2008). Annexin A1 regulates intestinal mucosal injury, inflammation, and repair.. J Immunol.

[8] Rajakariar R, Hilliard M, Lawrence T, Trivedi S, Colville-Nash P (2007). Hematopoietic prostaglandin D2 synthase controls the onset and resolution of acute inflammation through PGD2 and 15-deoxyDelta12 14 PGJ2.. Proc Natl Acad Sci U S A.

[9] Flower RJ, Rothwell NJ (1994). Lipocortin-1: cellular mechanisms and clinical relevance.. Trends Pharmacol Sci.

[10] Chatterjee BE, Yona S, Rosignoli G, Young RE, Nourshargh S (2005). Annexin 1-deficient neutrophils exhibit enhanced transmigration in vivo and increased responsiveness in vitro.. J Leukoc Biol.

[11] Mancuso F, Flower RJ, Perretti M (1995). Leukocyte transmigration, but not rolling or adhesion, is selectively inhibited by dexamethasone in the hamster post-capillary venule. Involvement of endogenous lipocortin 1.. J Immunol.

[12] Babbin BA, Laukoetter MG, Nava P, Koch S, Lee WY (2008). Annexin A1 regulates intestinal mucosal injury, inflammation, and repair.. J Immunol.

[13] Babbin BA, Lee WY, Parkos CA, Winfree LM, Akyildiz A (2006). Annexin I regulates SKCO-15 cell invasion by signaling through formyl peptide receptors.. J Biol Chem.

[14] Fiore S, Serhan CN (1990). Formation of lipoxins and leukotrienes during receptor-mediated interactions of human platelets and recombinant human granulocyte/macrophage colony-stimulating factor-primed neutrophils.. J Exp Med.

[15] Serhan CN, Sheppard KA (1990). Lipoxin formation during human neutrophil-platelet interactions. Evidence for the transformation of leukotriene A4 by platelet 12-lipoxygenase in vitro.. J Clin Invest.

[16] Bandeira-Melo C, Bozza PT, Diaz BL, Cordeiro RS, Jose PJ (2000). Cutting edge: lipoxin (LX) A4 and aspirin-triggered 15-epi-LXA4 block allergen-induced eosinophil trafficking.. J Immunol.

[17] Clish CB, Gronert K, Serhan CN (2000). Local and systemic delivery of an aspirin-triggered lipoxin stable analog inhibits neutrophil trafficking.. Ann N Y Acad Sci.

[18] Godson C, Mitchell S, Harvey K, Petasis NA, Hogg N (2000). Cutting edge: lipoxins rapidly stimulate nonphlogistic phagocytosis of apoptotic neutrophils by monocyte-derived macrophages.. J Immunol.

[19] Maddox JF, Serhan CN (1996). Lipoxin A4 and B4 are potent stimuli for human monocyte migration and adhesion: selective inactivation by dehydrogenation and reduction.. J Exp Med.

[20] Maddox JF, Serhan CN (1996). Lipoxin A4 and B4 are potent stimuli for human monocyte migration and adhesion: selective inactivation by dehydrogenation and reduction.. J Exp Med.

[21] Mitchell S, Thomas G, Harvey K, Cottell D, Reville K (2002). Lipoxins, aspirin-triggered epi-lipoxins, lipoxin stable analogues, and the resolution of inflammation: stimulation of macrophage phagocytosis of apoptotic neutrophils in vivo.. J Am Soc Nephrol.

[22] Colgan SP, Serhan CN, Parkos CA, Delp-Archer C, Madara JL (1993). Lipoxin A4 modulates transmigration of human neutrophils across intestinal epithelial monolayers.. J Clin Invest.

[23] Goh J, Baird AW, O'Keane C, Watson RW, Cottell D (2001). Lipoxin A(4) and aspirin-triggered 15-epi-lipoxin A(4) antagonize TNF-alpha-stimulated neutrophil-enterocyte interactions in vitro and attenuate TNF-alpha-induced chemokine release and colonocyte apoptosis in human intestinal mucosa ex vivo.. J Immunol.

[24] Gewirtz AT, McCormick B, Neish AS, Petasis NA, Gronert K (1998). Pathogen-induced chemokine secretion from model intestinal epithelium is inhibited by lipoxin A4 analogs.. J Clin Invest.

[25] Gronert K, Gewirtz A, Madara JL, Serhan CN (1998). Identification of a human enterocyte lipoxin A4 receptor that is regulated by interleukin (IL)-13 and interferon gamma and inhibits tumor necrosis factor alpha-induced IL-8 release.. J Exp Med.

[26] Perretti M, Chiang N, La M, Fierro IM, Marullo S (2002). Endogenous lipid- and peptide-derived anti-inflammatory pathways generated with glucocorticoid and aspirin treatment activate the lipoxin A4 receptor.. Nat Med.

[27] Babbin BA, Laukoetter MG, Nava P, Koch S, Lee WY (2008). Annexin A1 regulates intestinal mucosal injury, inflammation, and repair.. J Immunol.

[28] Gastardelo TS, Damazo AS, Dalli J, Flower RJ, Perretti M (2009). Functional and ultrastructural analysis of annexin A1 and its receptor in extravasating neutrophils during acute inflammation.. Am J Pathol.

[29] Gavins FN, Yona S, Kamal AM, Flower RJ, Perretti M (2003). Leukocyte antiadhesive actions of annexin 1.. Blood.

[30] Martin GR, Perretti M, Flower RJ, Wallace JL (2008). Annexin-1 modulates repair of gastric mucosal injury.. Am J Physiol Gastrointest Liver Physiol.

[31] Croxtall JD, Waheed S, Choudhury Q, Anand R, Flower RJ (1993). N-terminal peptide fragments of lipocortin-1 inhibit A549 cell growth and block EGF-induced stimulation of proliferation.. Int J Cancer.

[32] Croxtall JD, Choudhury Q, Flower RJ (1998). Inhibitory effect of peptides derived from the N-terminus of lipocortin 1 on arachidonic acid release and proliferation in the A549 cell line: identification of E-Q-E-Y-V as a crucial component.. Br J Pharmacol.

[33] Pederzoli-Ribeil M, Maione F, Cooper D, Al Kashi A, Dalli J (2010). Design and characterization of a cleavage-resistant Annexin A1 mutant to control inflammation in the microvasculature.. Blood.

[34] Serhan CN, Savill J (2005). Resolution of inflammation: the beginning programs the end.. Nat Immunol.

[35] Grisham MB (1994). Oxidants and free radicals in inflammatory bowel disease.. Lancet.

[36] Nusrat A, Parkos CA, Liang TW, Carnes DK, Madara JL (1997). Neutrophil migration across model intestinal epithelia: monolayer disruption and subsequent events in epithelial repair.. Gastroenterology.

[37] Rugtveit J, Nilsen EM, Bakka A, Carlsen H, Brandtzaeg P (1997). Cytokine profiles differ in newly recruited and resident subsets of mucosal macrophages from inflammatory bowel disease.. Gastroenterology.

[38] Rugtveit J, Haraldsen G, Hogasen AK, Bakka A, Brandtzaeg P (1995). Respiratory burst of intestinal macrophages in inflammatory bowel disease is mainly caused by CD14+L1+ monocyte derived cells.. Gut.

[39] Bystrom J, Evans I, Newson J, Stables M, Toor I (2008). Resolution-phase macrophages possess a unique inflammatory phenotype that is controlled by cAMP.. Blood.

[40] Vong L, Ferraz JG, Panaccione R, Beck PL, Wallace JL (2010). A pro-resolution mediator, prostaglandin D(2), is specifically up-regulated in individuals in long-term remission from ulcerative colitis.. Proc Natl Acad Sci U S A.

[41] Colgan SP, Serhan CN, Parkos CA, Delp-Archer C, Madara JL (1993). Lipoxin A4 modulates transmigration of human neutrophils across intestinal epithelial monolayers.. J Clin Invest.

[42] Goh J, Baird AW, O'Keane C, Watson RW, Cottell D (2001). Lipoxin A(4) and aspirin-triggered 15-epi-lipoxin A(4) antagonize TNF-alpha-stimulated neutrophil-enterocyte interactions in vitro and attenuate TNF-alpha-induced chemokine release and colonocyte apoptosis in human intestinal mucosa ex vivo.. J Immunol.

[43] Fiorucci S, Wallace JL, Mencarelli A, Distrutti E, Rizzo G (2004). A beta-oxidation-resistant lipoxin A4 analog treats hapten-induced colitis by attenuating inflammation and immune dysfunction.. Proc Natl Acad Sci U S A.

[44] Gewirtz AT, Collier-Hyams LS, Young AN, Kucharzik T, Guilford WJ (2002). Lipoxin a4 analogs attenuate induction of intestinal epithelial proinflammatory gene expression and reduce the severity of dextran sodium sulfate-induced colitis.. J Immunol.

[45] Lee TH, Crea AE, Gant V, Spur BW, Marron BE (1990). Identification of lipoxin A4 and its relationship to the sulfidopeptide leukotrienes C4, D4, and E4 in the bronchoalveolar lavage fluids obtained from patients with selected pulmonary diseases.. Am Rev Respir Dis.

[46] Pouliot M, Clish CB, Petasis NA, Van Dyke TE, Serhan CN (2000). Lipoxin A(4) analogues inhibit leukocyte recruitment to Porphyromonas gingivalis: a role for cyclooxygenase-2 and lipoxins in periodontal disease.. Biochemistry.

[47] Edenius C, Kumlin M, Bjork T, Anggard A, Lindgren JA (1990). Lipoxin formation in human nasal polyps and bronchial tissue.. FEBS Lett.

[48] Jupp J, Hillier K, Elliott DH, Fine DR, Bateman AC (2007). Colonic expression of leukotriene-pathway enzymes in inflammatory bowel diseases.. Inflamm Bowel Dis.

[49] Perretti M, Chiang N, La M, Fierro IM, Marullo S (2002). Endogenous lipid- and peptide-derived anti-inflammatory pathways generated with glucocorticoid and aspirin treatment activate the lipoxin A4 receptor.. Nat Med.

[50] Souza DG, Fagundes CT, Amaral FA, Cisalpino D, Sousa LP (2007). The required role of endogenously produced lipoxin A4 and annexin-1 for the production of IL-10 and inflammatory hyporesponsiveness in mice.. J Immunol.

[51] Brancaleone V, Dalli J, Bena S, Flower RJ, Cirino G (2011). Evidence for an Anti-Inflammatory Loop Centered on Polymorphonuclear Leukocyte Formyl Peptide Receptor 2/Lipoxin A4 Receptor and Operative in the Inflamed Microvasculature.. J Immunol.

[52] Vergnolle N, Pages P, Guimbaud R, Chaussade S, Bueno L (2004). Annexin 1 is secreted in situ during ulcerative colitis in humans.. Inflamm Bowel Dis.

[53] Babbin BA, Laukoetter MG, Nava P, Koch S, Lee WY (2008). Annexin A1 regulates intestinal mucosal injury, inflammation, and repair.. J Immunol.

[54] Babbin BA, Lee WY, Parkos CA, Winfree LM, Akyildiz A (2006). Annexin I regulates SKCO-15 cell invasion by signaling through formyl peptide receptors.. J Biol Chem.

[55] Martin GR, Perretti M, Flower RJ, Wallace JL (2008). Annexin-1 modulates repair of gastric mucosal injury.. Am J Physiol Gastrointest Liver Physiol.

[56] Babbin BA, Laukoetter MG, Nava P, Koch S, Lee WY (2008). Annexin A1 regulates intestinal mucosal injury, inflammation, and repair.. J Immunol.

[57] Vong L, D'Acquisto F, Pederzoli-Ribeil M, Lavagno L, Flower RJ (2007). Annexin 1 cleavage in activated neutrophils: a pivotal role for proteinase 3.. J Biol Chem.

[58] Duncan R, Carpenter B, Main LC, Telfer C, Murray GI (2008). Characterisation and protein expression profiling of annexins in colorectal cancer.. Br J Cancer.

[59] Jiang D, Ying W, Lu Y, Wan J, Zhai Y (2003). Identification of metastasis-associated proteins by proteomic analysis and functional exploration of interleukin-18 in metastasis.. Proteomics.

[60] Xin W, Rhodes DR, Ingold C, Chinnaiyan AM, Rubin MA (2003). Dysregulation of the annexin family protein family is associated with prostate cancer progression.. Am J Pathol.

[61] Perretti M, Chiang N, La M, Fierro IM, Marullo S (2002). Endogenous lipid- and peptide-derived anti-inflammatory pathways generated with glucocorticoid and aspirin treatment activate the lipoxin A4 receptor.. Nat Med.

[62] Ye RD, Boulay F, Wang JM, Dahlgren C, Gerard C (2009). International Union of Basic and Clinical Pharmacology. LXXIII. Nomenclature for the formyl peptide receptor (FPR) family.. Pharmacol Rev.

[63] Le Y, Murphy PM, Wang JM (2002). Formyl-peptide receptors revisited.. Trends Immunol.

[64] Babbin BA, Lee WY, Parkos CA, Winfree LM, Akyildiz A (2006). Annexin I regulates SKCO-15 cell invasion by signaling through formyl peptide receptors.. J Biol Chem.

[65] der Weid PY, Hollenberg MD, Fiorucci S, Wallace JL (2004). Aspirin-triggered, cyclooxygenase-2-dependent lipoxin synthesis modulates vascular tone.. Circulation.

[66] Gastardelo TS, Damazo AS, Dalli J, Flower RJ, Perretti M (2009). Functional and ultrastructural analysis of annexin A1 and its receptor in extravasating neutrophils during acute inflammation.. Am J Pathol.

[67] Gavins FN, Yona S, Kamal AM, Flower RJ, Perretti M (2003). Leukocyte antiadhesive actions of annexin 1.. Blood.

[68] Martin GR, Perretti M, Flower RJ, Wallace JL (2008). Annexin-1 modulates repair of gastric mucosal injury.. Am J Physiol Gastrointest Liver Physiol.

[69] Scannell M, Flanagan MB, deStefani A, Wynne KJ, Cagney G (2007). Annexin-1 and peptide derivatives are released by apoptotic cells and stimulate phagocytosis of apoptotic neutrophils by macrophages.. J Immunol.

[70] Souza DG, Fagundes CT, Amaral FA, Cisalpino D, Sousa LP (2007). The required role of endogenously produced lipoxin A4 and annexin-1 for the production of IL-10 and inflammatory hyporesponsiveness in mice.. J Immunol.

[71] Dufton N, Hannon R, Brancaleone V, Dalli J, Patel HB (2010). Anti-inflammatory role of the murine formyl-peptide receptor 2: ligand-specific effects on leukocyte responses and experimental inflammation.. J Immunol.

[72] Gronert K, Gewirtz A, Madara JL, Serhan CN (1998). Identification of a human enterocyte lipoxin A4 receptor that is regulated by interleukin (IL)-13 and interferon gamma and inhibits tumor necrosis factor alpha-induced IL-8 release.. J Exp Med.

[73] Vong L, Ferraz JG, Panaccione R, Beck PL, Wallace JL (2010). A pro-resolution mediator, prostaglandin D(2), is specifically up-regulated in individuals in long-term remission from ulcerative colitis.. Proc Natl Acad Sci U S A.

[74] Chavez-Pina AE, Vong L, McKnight W, Dicay M, Zanardo RC (2008). Lack of effects of acemetacin on signalling pathways for leukocyte adherence may explain its gastrointestinal safety.. Br J Pharmacol.

[75] Vong L, Ferraz JG, Panaccione R, Beck PL, Wallace JL (2010). A pro-resolution mediator, prostaglandin D(2), is specifically up-regulated in individuals in long-term remission from ulcerative colitis.. Proc Natl Acad Sci U S A.

[76] Perretti M, Chiang N, La M, Fierro IM, Marullo S (2002). Endogenous lipid- and peptide-derived anti-inflammatory pathways generated with glucocorticoid and aspirin treatment activate the lipoxin A4 receptor.. Nat Med.

[77] Souza MH, de Lima OM, Zamuner SR, Fiorucci S, Wallace JL (2003). Gastritis increases resistance to aspirin-induced mucosal injury via COX-2-mediated lipoxin synthesis.. Am J Physiol Gastrointest Liver Physiol.

